# Influence of perceived social support on detection of social norm violation: evidence from N1 and N400

**DOI:** 10.3389/fpsyg.2024.1336186

**Published:** 2024-02-28

**Authors:** Bing Liang, Bingbing Li, Xiaoyue Fan, Yan Mu, Juan Wang

**Affiliations:** ^1^College of Education Science, Jiangsu Normal University, Xuzhou, China; ^2^CAS Key Laboratory of Behavioral Science,Institute of Psychology Chinese Academy of Sciences, Beijing, China; ^3^Department of Psychology, University of Chinese Academy of Sciences, Beijing, China

**Keywords:** perceived social support, norm violation, social norms, N1, N400

## Abstract

**Introduction:**

The perceived social support individuals receive from their others plays a crucial role in shaping conformity with social norms. However, the specific mechanism underlying perceived social support and the detection of social norms remains unclear.

**Methods:**

In this study, college students with high and low levels of perceived social support were asked to judge the appropriateness of stranger’s behaviors (e.g., singing) in different situations (e.g., library). The participants’ electroencephalography activities were analyzed aiming to uncover the neural mechanism underlying how perceived social support influences the detection of others’ normative behavior.

**Results:**

The ERP results indicate that, for individuals with a lower level of perceived social support, larger amplitudes of the N1 component (related to primary processing) and the N400 component (related to cognitive conflict) were observed when detecting others’ social norm violation compared to the conformity condition. However, for individuals with a higher level of perceived social support, no significant differences were found in detecting others’ conformity or violation of social norms.

**Discussion:**

The results indicate that, when the perceived social support level of the individual is low, detecting others’ social norm violation elicits deeper primary processing and stronger cognitive conflict compared to conformity condition.

## Introduction

Social norms are not explicitly stated (Bicchieri, 2006), but rather rely on individuals’ understanding and interpretation of what is considered appropriate or inappropriate behavior. Although the enforcement of social norms is universal, there is wide variation in the strength of social norms across human groups (Mu et al., 2015). The seminal research in social neuroscience has shown that the N400 can serve as a powerful neurophysiological marker for detecting unexpected anomalous stimuli and affective and social incongruent information (White et al., 2009; Na and Kitayama, 2011). Mu et al. (2015) recorded EEG activity from Chinese and US participants during a social norm violation task where they judged the appropriateness of certain behaviors in given situations. They observed increased N400 activity in the central and parietal regions, supporting the detection of norm violations and suggesting N400 as a unique neural marker for social norm violation detection. This study suggests that the N400 effect reflects a cognitive conflict experienced by individuals when perceiving others engaging in inappropriate behavior within specific social contexts. Subsequently, a series of studies further confirmed that the N400 is indeed an indicator of social violation (Luo et al., 2019; Salvador et al., 2020a,b; Goto et al., 2022). Research generally suggests that the N1 component reflects rapid and automatic intuition processes during decision-making, representing the primary processing of relevant information such as goodness, badness, truth, and falsehood (Scheele et al., 2014; Gui et al., 2016). Studies have also shown that N1 can distinguish between good and bad moral behaviors, and immoral behaviors elicit a larger N1 amplitude than moral behaviors (Yoder and Decety, 2014; Gui et al., 2016). The detection process of social norms additionally incorporates the evaluation of valence information, distinguishing between good or bad detection and true or false detection. This study aims to investigate whether there is a distinct distribution of the N1 component in the detection of social norms.

Perceived social support refers to an individual’s subjective perception of the assistance and support received from others (Dunkel-Schetter and Bennett, 1990). Perceived social support and social norms are not only expected to influence, independently, a person’s willingness to judgment, but also in interaction with each other. Cullum et al. suggested that the social support individuals perceived from peers may be a significant factor in their conformity to social norm (Cullum et al., 2013). That is, perceived social support will affect the satisfaction of individuals’ affiliation needs. When people’s affiliation needs are threatened or left unfulfilled, they should be more motivated to attend to social cues such as normative behavior (Lakin and Chartrand, 2003; Cullum et al., 2011), and more receptive to social influences on their behavior (Lakin and Chartrand, 2003) as a result of efforts to improve their rapport with others by conforming to socially approved and typical behavior. Despite previous findings indicating that individuals with low perceived social support are more susceptible to the influence of social norms (Cullum et al., 2011), the underlying mechanism by which perceived social support influences the detection of others’ social norm behavior remains unclear.

It can be seen that there are limitations in current research on social norms. On the one hand, the strength of social norms may vary significantly among different human groups, and more exploration is needed to understand the reasons for these differences. On the other hand, the underlying mechanisms through which perceived social support influences the detection of others’ social norm behavior are still unclear and require further research. Based on the results of previous studies (Lakin and Chartrand, 2003; Taylor and Lonsdale, 2010; Cullum et al., 2011, 2013), this study hypothesizes that perceived social support influences detection of social norm violation. If individuals with a low level of perceived social support have greater N1 amplitude when detecting social norm violation, but individuals with a high level of perceived social support have no difference in N1 amplitude when detecting social norm violation and conformity, it indicates that perceived social support influences primary processing when detecting others’ social norm violations. Meanwhile, if individuals with a low level of perceived social support have greater N400 amplitude when detecting social norm violation, but individuals with a high level of perceived social support have no difference in N400 amplitude when detecting social norm violation and conformity, it indicates that perceived social support influences cognitive conflict when detecting others’ social norm violations. The main purpose of this study is to enhance our understanding of how perceived social support affects the detection of social norm violation, as well as to investigate the underlying mechanisms involved in this process.

## Methods

### Participants

The *Perceived Social Support Scale* was used to measure 422 college students. Although participants were recruited through online random distribution of questionnaires, upon arrival at the laboratory, eligible participants underwent a secondary questionnaire to eliminate the possibility of participants not taking the online questionnaire seriously. Participants were categorized into the low or high perceived social support group based on their questionnaire scores, with the top 27% or bottom 27% of scores representing the high or low perceived social support group, respectively. The repeated measures ANOVA with 34 participants would be sensitive to medium effects (*f* = 0.25) (using G^*^Power SPSS calculation, Faul et al., 2009) with 80% power (alpha = 0.05) for main effects and interactions. A total of 28 participants were selected from the low and high perceived social support groups, respectively, and 4 participants were excluded due to excessive EEG artifacts. Finally, a total of 52 participants were counted in the data analysis. Among them, there were 26 participants in the low perceived social support group (mean age: 20.81 ± 2.47 years, 17 females), and 26 participants in the high perceived social support group (mean age: 21.42 ± 2.94 years, 12 females). Independent sample *t* test showed that there were significant differences in the scores of perceived social support between the low and high perceived social support groups [*t* (50) = −21.376, *p* < 0.001]. All participants were right-handed, had normal vision or corrected vision, and had no history of mental illness or head trauma. All participants gave written informed consent, and this study was approved by the Department of Psychology of Jiangsu Normal University Ethics Committee.

### Experimental design

The experiment employed a mixed design of 2 (perceived social support: high vs. low) x 2 (social norm: conformity vs. violation), in which perceived social support was a between-subject variable and social norm was a within-subject variable. The dependent variables were the accuracy of responses, response times (RTs), and the two ERP components that related to the detection of social norm violations (i.e., N1 and N400).

### Materials

Adapted from previous research (Mu et al., 2015), 53 sets of self-designed experimental materials were compiled. Each set of sentences included two behavior x situation combinations, that is, compliance with social norms and violation of social norms. For example, a sentence that conforms to social norms (e.g., Xiao Hong is in the concert hall. She is singing.), and a sentence that violates social norms (e.g., Xiao Hong is in the library. She is singing.). During the preparation phase of the experiment, we also assessed the valence of behaviors in the experimental materials. We used a self-developed “Behavioral Moral Color Rating Questionnaire” to randomly select 30 college students to assess whether the sentences presented exhibited social moral value, and to note any difficulties or uncertainties they encountered during the evaluation process. After the evaluation, a total of 17 biased behaviors and repeated occurrences were removed, resulting in 53 behaviors that were retained as materials for the subsequent formal experimental study. Before the EEG experiment, we recruited 51 college students to rate the degree to which behaviors violated social norms in the experimental materials using a 7-point Likert Scale (1 = strongly conforming, 7 = strongly violating). The results of independent sample *t*-test analysis showed that the scores of statements of violations of social norms (*M* = 6.67, *SD* = 0.82) and statements of conformity to social norms (*M* = 1.46, *SD* = 1.01) in each set of materials were significantly different [*t* (1, 52) = 209.18, *p* < 0.001], validating the adapted social norm materials.

### Procedure

**Figure 1:**
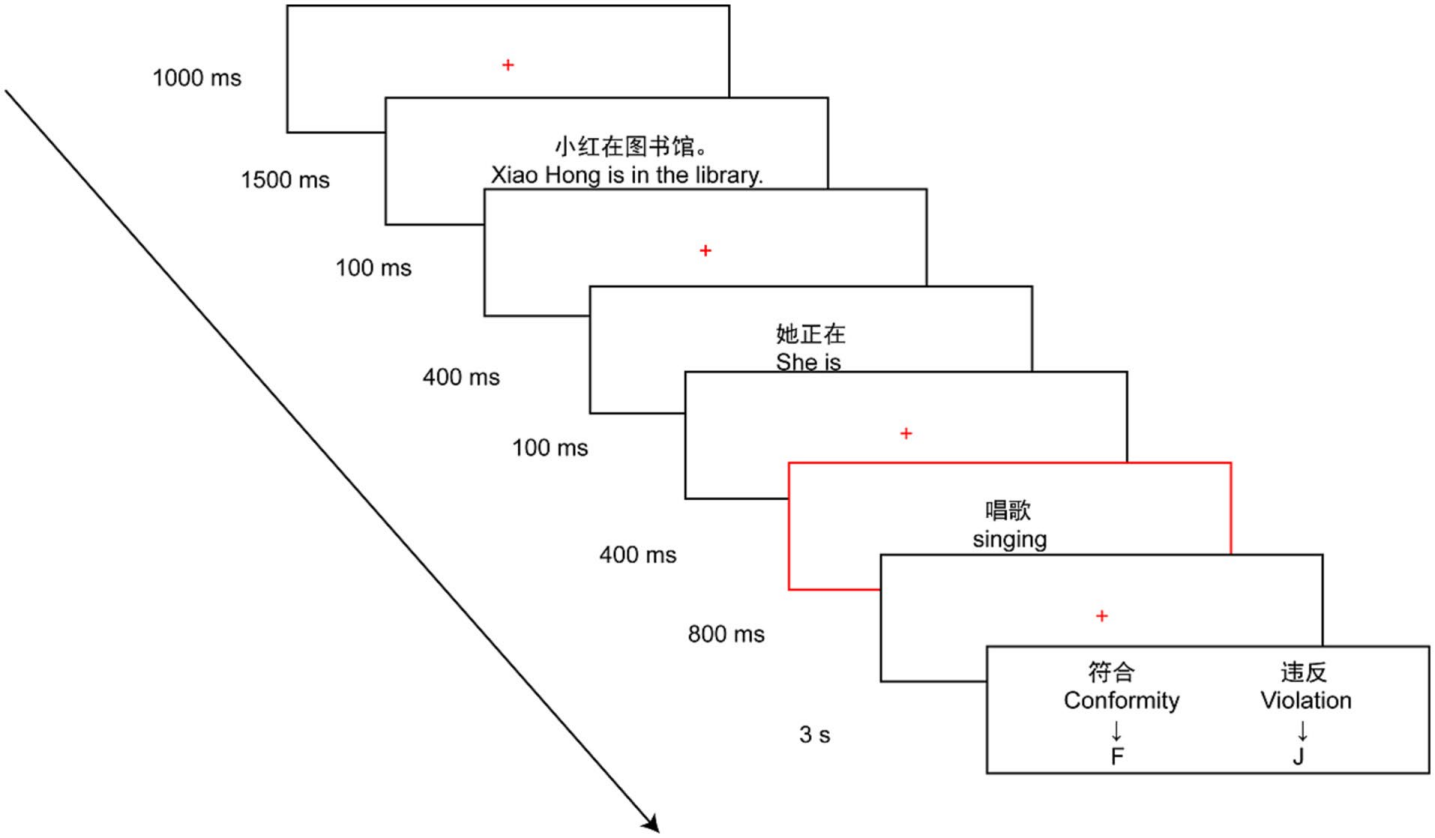
The procedure of social norm violation task. Participants were asked to judge whether certain behavior is appropriate or not in a given situation. Each behavior (e.g., singing) was set in two situations: conformity conditions (e.g., concert hall), and violation conditions (e.g., library). Participants were asked to use their index fingers on the left and right hand to judge whether the behavior was appropriate, using the F (conformity) and J (violation) keys on the keyboard. The rectangle with a red frame is the crucial word (e.g., singing) for generating ERP components.

Participants were asked to complete a social norm violation task consisting of 53 behaviors. A total of 106 trials were randomly assigned to 4 blocks, with each block lasting about 4 min. The experimental stimuli were developed and administered using E-Prime 3.0 software (Psychology Software Tools, Sharpsburg, Maryland, United States). As shown in Figure 1, each trial started with 1,000 ms of fixation. Thereafter, the first sentence depicting the situation was presented for 1,500 ms (e.g., Xiao Hong is in the library), followed by a fixation of 100 ms. Then the second sentence depicted a specific behavior (e.g., she is singing), which was separated into two successive 400 ms screens with a 100 ms fixation (e.g., She is was shown for 400 ms, followed by a 100 ms fixation and then singing was shown for 400 ms). After an 800 ms fixation, a response screen was shown for 3 s (at a viewing distance of 70 cm). During this period, the participants were asked to judge the appropriateness of the behavior while ensuring the correctness and press the key as quickly as possible. Both index fingers were placed on the keys F (conformity) and J (violation) on the keyboard during the period of the experiment. ERP components were generated on a screen with a red frame (e.g., singing). The response buttons were counterbalanced across participants. The experiment was conducted in a soundproof room with constant lighting and comfortable temperature. During the whole experiment, the participants were asked to watch the center of the screen, keep their bodies relaxed, and minimize head movement, blinking, swallowing, and other actions.

### Data recordings and analyses

A 64-channel electroencephalogram (EEG) recording system (Brain Product, Germany) was utilized to record and analyze the ERPs. EEG was amplified (bandpass 0.01–100 Hz) and digitized at a sampling rate of 1,000 Hz. The vertical electrooculogram (VEOG) was recorded from two electrodes located above and below the left eye. The horizontal electrooculogram (HEOG) was recorded from the two electrodes placed 1.5 cm lateral to the left and right external canthi. The right mastoid was used as a reference during online recording, and all data were re-referenced offline to an average mastoid reference. The impedance of the electrodes during the recording was below 5 kΩ. EEGs were segmented beginning 200 ms before the onset of the critical action stimulus and ending 1,000 ms after its onset. The rectangle with a red frame is the crucial action (e.g., singing) for generating ERP components. EEGlab and ERPlab were used for the offline analysis (Delorme and Makeig, 2004; Lopez-Calderon and Luck, 2014). During the offline analysis, EEG was treated with band-pass filtering (0.1–30 Hz). Continuous data were algorithmically corrected for the possible artifacts (eye movements and blinks, cardiac signals, muscle noise, and line noise) by independent component analysis (ICA). The corrected data were epoched into a 1,200 ms time window with a 200 ms prestimulus baseline in the social norm violation task, consistent with previous research (Mu et al., 2015; Luo et al., 2019). Epochs that exceeded ±60 μV were omitted from the average. Across conditions, the mean trial accept rate was 95.86%, with no significant difference between conditions (low perceived social support + conformity: 96.70%, low perceived social support + violation: 96.18%, high perceived social support + conformity: 95.61%, high perceived social support + violation: 94.94%).

For ERP data, the time windows for N1 and N400 were determined based on previous ERP studies (Mu et al., 2015; Luo et al., 2019; Goto et al., 2022; Zhan et al., 2022) and the visual observation on the grand average ERPs of the current study. Specifically, the time windows were set as and 80–160 ms for N1and 250–450 ms for N400. ERP data were analyzed with ANOVA with factors: 2 (perceived social support: high vs. low) x 2 (social norm: conformity vs. violation). The statistical analysis was conducted using SPSS 28.0. The *p*-values for main and interaction effects were corrected using the Greenhouse–Geisser method for violations of the sphericity assumption. With reference to the previous ERPs study, the following six electrode clusters were constructed for ERP analysis: anterior-frontal (AF): AF3, AF7, AFz, AF4, and AF8; frontal (F): F1, F3, F5, F7, Fz, F2, F4, F6, F8, FC1, FC3, FC5, FCz, FC2, FC4, and FC6; temporal (T): T7, T8, TP7, TP8, FT7, and FT8; central (C): C1, C3, Cz, C2, and C4; central-parietal (CP): CP1, CP3, CPz, CP2, and CP4; parietal (P): P1, P3, Pz, P2, P4, PO3, PO7, POz, PO4, and PO8. The mean amplitudes of the N1 and N400 components within the time windows of 80–160 ms and 250–450 ms, respectively, were averaged across all electrodes within each cluster to obtain the mean amplitudes of ERPs for each cluster. The mean amplitudes of the ERPs at each cluster under each condition were then entered into further analysis.

## Results

### Behavioral results

We conducted a 2 (perceived social support: low, high) × 2 (social norm: conformity, violation) ANOVA on the accuracy and reaction times of social norm task judgment, and the results showed that the main effect of perceived social support, the main effect of social norms and the interaction between perceived social support and social norms were not significant (*p*s > 0.05).

### Event-related potential results

#### N1 (80–160 ms)

**Figure 2:**
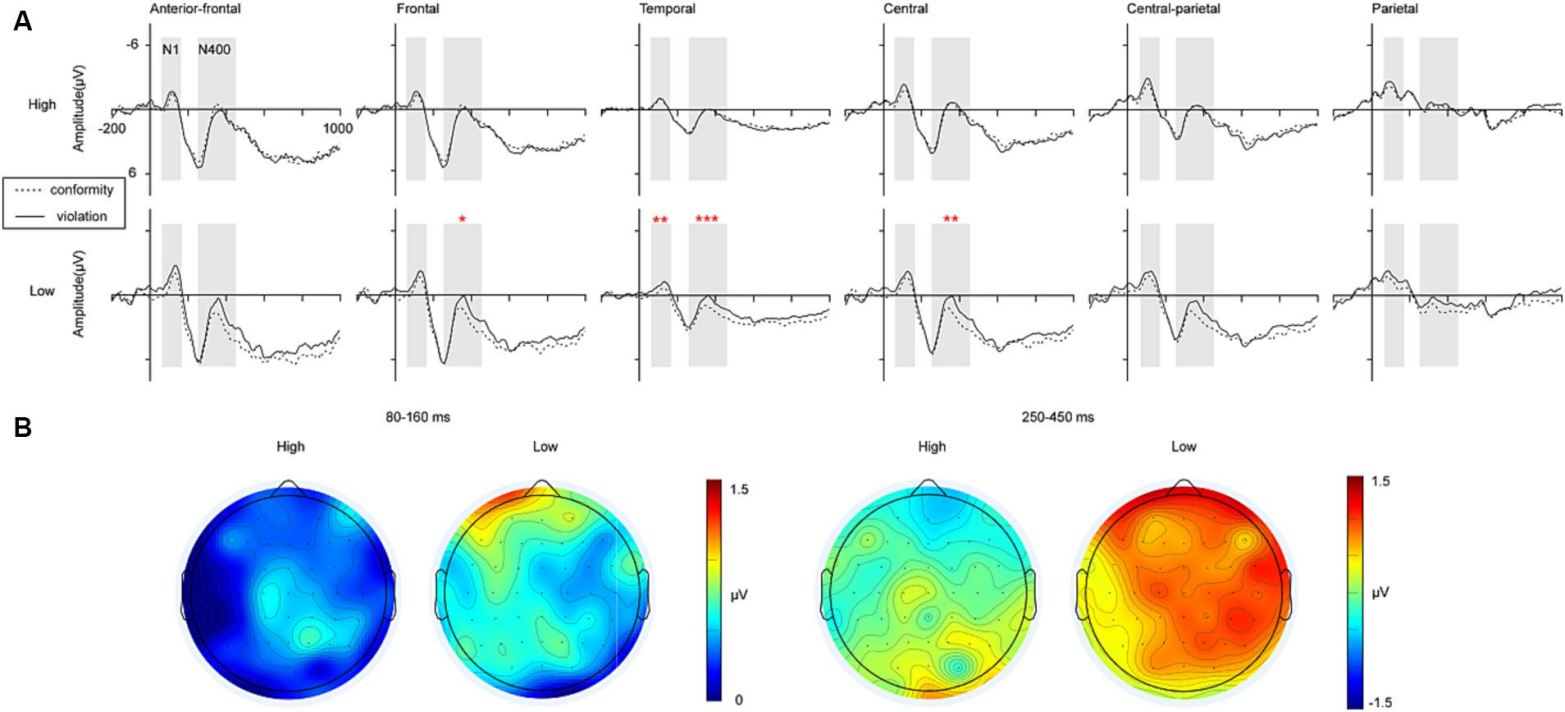
Waveforms and topographic maps. **(A)** Grand average event-related potentials (ERPs) of social norm violation task in the anterior-frontal, frontal, temporal, central, central-parietal, and parietal regions. The shaded 80–160 ms time window was used for the calculation of the average amplitudes of the N1. The shaded 250–450 ms time window was used for the calculation of the average amplitudes of the N400. **(B)** Topographic maps for the differences of N1 and N400 in the 80–160 and 250–450 ms time window (conformity – violation). HIGH, the high interpersonal intimacy; LOW, the low interpersonal intimacy.

A 2 (perceived social support: low, high) ×2 (social norm: conformity, violation) ANOVA of N1 in the anterior-frontal, frontal, temporal, central, central-parietal, and parietal regions was conducted. The main effect of social norm was significant in all regions, and social norm violation elicited greater N1 amplitudes than conformity condition (*p*s < 0.030). The main effect of perceived social support was not significant in all regions (*p*s > 0.05). The interaction between perceived social support and social norm was significant in the temporal region [temporal: *F* (1, 50) = 4.325, *p* = 0.043, *η^2^_p_* = 0.080]. As shown in Figure 2, simple effect analysis of the data from the temporal region found that for the low perceived social support group, the N1 amplitudes elicited by violating social norm were greater than those elicited by conforming to social norms [temporal: *F* (1, 50) = 10.639, *p* = 0.002, *η^2^_p_* = 0.114]. For the high perceived social support group, there were no significant differences in the N1 amplitudes elicited by violating social norms and conforming to social norms [temporal: *F* (1, 50) = 0.103, *p* = 0.750, *η^2^_p_* = 0.002].

#### N400 (250–450 ms)

**Figure 3:**
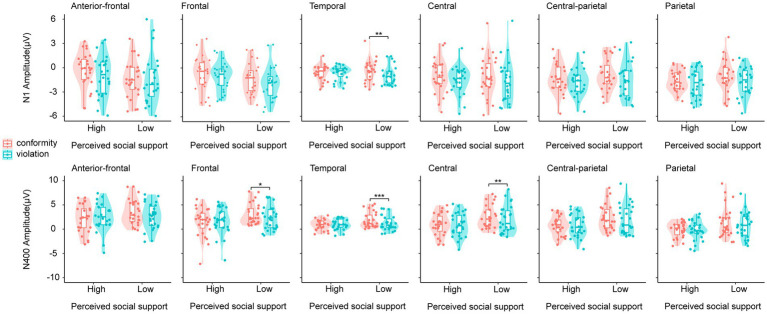
The mean amplitudes of N1 and N400 in the anterior-frontal/frontal/temporal/central/central-parietal/parietal region. High, the high interpersonal intimacy; Low, the low interpersonal intimacy. ^*^p < 0.05, ^**^p < 0.01, and ^***^p < 0.001, respectively.

A 2 (perceived social support: low, high) ×2 (social norm: conformity, violation) ANOVA of N400 in the anterior-frontal, frontal, temporal, central, central-parietal, and parietal regions was conducted. The main effect of perceived social support was significant in the central and central-parietal regions, and the high perceived social support group elicited greater N400 amplitudes than the low perceived social support group [central: *F* (1, 50) = 4.777, *p* = 0.034, *η^2^_p_* = 0.087; central-parietal: *F* (1, 50) = 4.747, *p* = 0.034, *η^2^_p_* = 0.087]. The main effect of social norm was significant in the temporal, central, and parietal regions, and social norm violation elicited greater N400 amplitudes than conformity condition [temporal: *F* (1, 50) = 4.378, *p* = 0.042, *η^2^_p_* = 0.081; central: *F* (1, 50) = 6.207, *p* = 0.016, *η^2^_p_* = 0.110; parietal: *F* (1, 50) = 6.565, *p* = 0.013, *η^2^_p_* = 0.116]. The interaction between perceived social support and social norm was significant in the frontal, temporal and central regions [frontal: *F* (1, 50) = 4.873, *p* = 0.032, *η^2^_p_* = 0.089; temporal: *F* (1, 50) = 7.846, *p* = 0.007, *η^2^_p_* = 0.136; central: *F* (1, 50) = 5.074, *p* = 0.029, *η^2^_p_* = 0.092]. As shown in Figure 3, simple effect analysis of the data from anterior-frontal, frontal, temporal and central regions found that for the low perceived social support group, the N400 amplitudes elicited by violating social norm were greater than those elicited by conforming to social norms [frontal: *F* (1, 50) = 6.175, *p* = 0.016, *η^2^_p_* = 0.110; temporal: *F* (1, 50) = 11.973, *p* < 0.001, *η^2^_p_* = 0.191; central: *F* (1, 50) = 11.253, *p* = 0.002, *η^2^_p_* = 0.161]. For the high perceived social support group, there were no significant differences in the N400 amplitudes elicited by violating social norms and conforming to social norms (*p*s > 0.05).

#### Correlation analysis between perceived social support score and differential ERPs

To further elucidate the relationship among perceived social support scores, N1 and N400, we conducted a correlation analysis perceived social support scores, differential N1, and differential N400. As shown in Table 1, correlation analysis showed that perceived social support was negatively correlated with differential N400 at the temporal, center-parietal and parietal regions. The perceived social support score was not significantly associated with differential N400 at other regions and differential N1 at all regions. That is, the lower the perceived social support score, the greater the differential N400 at the temporal, center-parietal and parietal regions.

**Table 1:** Correlation analysis between perceived social support score and differential ERPs.

Perceived social support score	Anterior frontal	Frontal	Temporal	Central	Central-parietal	Parietal
Differential N1	−0.086	−0.105	−0.255	−0.167	−0.143	−0.077
Differential N400	0.057	−0.101	−0.309^*^	−0.264	−0.362^**^	−0.336^*^

^*^ Differential ERPs = |conformity_ERP_ − violation_ERP_|. *p < 0.05, **p < 0.01.

## Discussion

In this study, we employed event-related potentials (ERPs) technology, which offers millisecond-level temporal resolution, to explore the influence of perceived social support on the detection of social norm violation and the time course of this cognitive process.

The behavioral results showed that the level of perceived social support had no significant effect on the accuracy rate and response time of social norm detection. Regardless of their level of perceived social support, participants were able to accurately detect whether behaviors conformed to or violated social norms. The ERP results indicate that, for individuals with a lower level of perceived social support, larger amplitudes of the N1 component (related to primary processing) and the N400 component (related to cognitive conflict) were observed when detecting others’ social norm violation compared to the conformity condition. However, for individuals with a higher level of perceived social support, no significant differences were found in detecting others’ conformity or violation of social norms. These results indicate that perceived social support influences detection of others’ social norms. It is shown the lower the level of perceived social support, the deeper the primary processing and stronger cognitive conflict will be elicited when the individual detect of social norm violation than conformity condition. It is of note that the magnitude of the temporal, central-parietal, and parietal differential N400 were related to perceived social support. The results showed that a lower level of perceived social support was associated with greater differential N400 at the temporal, central-parietal and parietal regions. This study offers empirical evidence to elucidate the temporal characteristics of brain processing influenced by perceived social support in detecting others’ social norm violation.

During the early stage, for individuals with a low level of perceived social support, the N1 amplitude for social norm violation was larger compared to conformity condition. These findings suggest that there exists a rapid and automatic intuitive process during the early stages of social norm violation detection, which is capable of deep and rapid primary processing of information relevant to decision making in the context (Gui et al., 2016). Building on previous research that found N1 can distinguish between good and bad moral behaviors (Yoder and Decety, 2014; Gui et al., 2016), we further found that for individuals with a low level of perceived social support, N1 can also differentiate between conformity and violation behaviors. For individuals with a low level of perceived social support receive less social support from family, friends and other important people, and cannot meet their affiliation needs. These individuals may attempt to improve their rapport with others by conforming to socially approved and typical behavior (Cullum et al., 2013) and they are more motivated to attend to social cues such as normative behavior (Lakin and Chartrand, 2003; Cullum et al., 2011). Normative information plays a crucial role in guiding their behaviors. Hence, individuals with a lower level of perceived social support conducted deeper primary processing of decision-making relevant information and automatically captured more attention in the detection of social norms. During the late stage, individuals with a lower level of perceived social support exhibited larger N400 amplitudes in response to social norm violation compared to the conformity condition. This may reflect a cognitive conflict that arises due to a mismatch between normative expectations and behaviors. In other words, the inconsistency between expected and observed behavior in a given situation could elicit a larger N400 amplitude, indicating a stronger cognitive conflict in the process of detecting social norm violation. Our research supports the N400 as a neural indicator of social norm violations (Mu et al., 2015; Luo et al., 2019; Salvador et al., 2020a,b; Goto et al., 2022). The N400 research on norm violations adds to the large body of research demonstrating the ubiquitous sensitivity of the N400 to incongruities across a variety of domains such as language, visual scenes, actions, memory, mathematics, and affect (Kutas and Federmeier, 2011), and it is consistent with the N400 as an ERP component reflective of a prediction error signal of meaning (Bornkessel-Schlesewsky and Schlesewsky, 2019).

No matter whether in the early stage or late stage, individuals with high perceived social support showed no significant difference in the ERP amplitudes elicited when detecting others’ conformity or violation of social norms. Individuals with a high level of perceived social support receive greater social support from their family, friends, and other significant individuals, which contributes to the fulfillment of their affiliation needs (Taylor and Lonsdale, 2010). Therefore, individuals with a high level of perceived social support may not feel the need to make changes to improve their relationships with others. Consequently, they may show less concern with regards to social norm violation compared to those with a low level of perceived social support. In addition, when individuals receive more social support, tend to have a more positive attitude towards others (Stinglhamber et al., 2006). Social norm violation involves damaging the rights and interests of others, which can serve as a negative stimulus that influences an individual’s decision-making process. Perceived social support serves as a protective mechanism for individuals, buffering the negative impact of adverse stimuli (Etzion, 1984; Fried and Tiegs, 1993). Therefore, when individuals with a high level of perceived social support detect others’ social norm violation, their perception of social support plays a protective role, which may be manifested as trying to reduce the extent of the behavior or seeking alternative explanations for it, and cannot help but reduce the improper degree of the behavior, so that there is no obvious differences between social norm violation and conformity. Moreover, there was a negative correlation between differential N400 and perceived social support, suggesting that individuals with a high level of perceived social support exhibited reduced sensitivity in detecting distinctions between social norm violation and conformity.

The experimental findings at the neural physiological level shed light on the impact of perceived social support among college students on the detection of social norm violation. Individuals with a low level of perceived social support tend to be more sensitive to social norms as they strive to fulfill their affiliation needs. Consequently, they exhibit heightened sensitivity and stronger responses towards social norm violation, as opposed to conformity condition. Individuals with a high level of perceived social support tend to hold a more positive attitude toward society due to they get greater support. They are more inclined to exhibit efforts to mitigate the severity of oyhers’ behavior or seek alternative explanations for it. Hence, when it comes to detecting social norm violation, there is no obvious neural physiological feedback associated with conformity to social norms. This distinctive pattern of physiological feedback may be closely linked to the satisfaction of affiliation needs and the protective effect of perceived social support.

The contributions of this study mainly include the following three aspects: Firstly, it illustrates the direct impact of perceived social support on social norm detections. Individuals with a lower level of perceived social support tend to perceive less support from others, organizations, and social networks. This lack of support hinders the fulfillment of their affiliation needs, leading them to pay greater attention to social cues (Lakin and Chartrand, 2003; Cullum et al., 2011). This study has enriched the study of social norm. Secondly, it extends previous research on the neural mechanisms through which perceived social support influences the evaluation of others’ social norms, and it provides further insights into the characteristics of detecting other people’s social normative behavior. Thirdly, this study extends the analytical perspective beyond the traditional focus on external environmental factors by examining the impact of individual social cognition on the assessment of others’ social norms. It emphasizes the importance of perceived social support in influencing individuals’ cognitive processes and decision-making related to social norm violations. Overall, this study contributes to the understanding of the influence of perceived social support on the detection and evaluation of social norm violations, providing valuable insights into social cognition.

This study has some limitations that provide suggestions for future research. On the one hand, our reliance on college students who are highly steeped in similar norms on campus, in academia, and on social media likely underestimates differences in the broader population. Therefore, data derived from college students may not fully represent the broader community. Future research should aim to include a more diverse range of participants to enhance the generalizability of the findings and improve our understanding of the research. On the other hand, individuals with high perceived social support tend to exhibit an “equal treatment” attitude towards others’ conformity or violation of social norms, whereas those with low perceived social support tend to display a “differential” attitude. Investigating the impact of these attitude differences on individuals’ compliance with social norms represents an important aspect for further research in the future.

## Conclusion

In sum, social norm detection plays a critical role in the development of human society and individuals’ social development. This study examined the influence of perceived social support on detecting others’ social norm violations, and revealed the temporal dynamics of brain activity behind it through event-related potential technology. Individuals with a lower level of perceived social support elicit larger amplitudes of N1 and N400 components when detecting others’ social norm violations. These neural markers are be associated with primary processing and cognitive conflict. When individuals have a lower level of perceived social support, detecting others’ social norm violations compared to conforming behavior elicits deeper levels of primary processing and stronger cognitive conflict. However, when individuals have a higher level of perceived social support, there is no difference in the primary processing and cognitive conflict elicited when detecting others’ conforming or violating social norm behavior.

## Data availability statement

The raw data supporting the conclusions of this article will be made available by the authors, without undue reservation.

## Ethics statement

The studies involving humans were approved by Ethics Committee, Jiangsu Normal University, China. The studies were conducted in accordance with the local legislation and institutional requirements. The participants provided their written informed consent to participate in this study.

## Author contributions

BLia: Conceptualization, Data curation, Formal analysis, Funding acquisition, Investigation, Methodology, Resources, Software, Validation, Visualization, Writing – original draft, Writing – review & editing, Project administration. BLi: Conceptualization, Methodology, Resources, Supervision, Validation, Writing – review & editing, Software. XF: Conceptualization, Methodology, Resources, Supervision, Writing – review & editing, Funding acquisition, Visualization. YM: Conceptualization, Methodology, Supervision, Writing – review & editing, Formal analysis, Project administration, Resources. JW: Conceptualization, Formal analysis, Methodology, Project administration, Resources, Supervision, Validation, Writing – review & editing, Funding acquisition.
